# Hypermetabolic lymphadenopathy following administration of BNT162b2 mRNA Covid-19 vaccine: incidence assessed by [^18^F]FDG PET-CT and relevance to study interpretation

**DOI:** 10.1007/s00259-021-05314-2

**Published:** 2021-03-27

**Authors:** Dan Cohen, Shir Hazut Krauthammer, Ido Wolf, Einat Even-Sapir

**Affiliations:** 1 Department of Nuclear Medicine, Tel-Aviv Sourasky Medical Center, 6 Weizmann St, 6423906 Tel Aviv, Israel; 2 Institute of Oncology, Tel-Aviv Sourasky Medical Center, 6 Weizmann St, 6423906 Tel Aviv, Israel; 3 Sackler Faculty of Medicine, Tel Aviv University, Tel Aviv, Israel

**Keywords:** Covid-19, Vaccination, False-positive [^18^F]FDG uptake, Axillary lymph nodes, Oncologic imaging

## Abstract

**Purpose:**

Nationwide mass vaccination against Covid-19 started in Israel in late 2020. Soon we identified on [^18^F]FDG PET-CT studies vaccine-associated hypermetabolic lymphadenopathy (VAHL) in axillary or supraclavicular lymph nodes (ASLN) ipsilateral to the vaccination site. Sometimes, differentiation between the malignant and benign nature of the hypermetabolic lymphadenopathy (HLN) could not be made, and equivocal HLN (EqHL) was reported. The purpose of the study was to determine the overall incidence of VAHL after BNT162b2 vaccination and also its relevance to PET-CT interpretation in oncologic patients.

**Methods:**

A total of 951 consecutive patients that underwent [^18^F]FDG PET-CT studies in our department were interviewed regarding the sites and dates of the vaccine doses. A total of 728 vaccinated patients (All-Vac group) were included: 346 received the first dose only (Vac-1 group) and 382 received the booster dose as well (Vac-2 group). Studies were categorized as no HLN, malignant-HLN (MHL), VAHL, or EqHL. In studies with VAHL, location, [^18^F]FDG-intensity uptake and nodes size were recorded.

**Results:**

The incidences of HLN were 45.6%, 36.4%, and 53.9% in All-Vac, Vac-1, and Vac-2 groups, respectively. VAHL was reported in 80.1% of vaccinated patients with HLN. Lower incidences of VAHL were found during the first 5 days or in the third week after the first vaccine and beyond 20 days after the booster dose. In 49 of 332 (14.8%) vaccinated patients, we could not determine whether HLN was MHL or VAHL. Breast cancer and lymphoma were the leading diseases with EqHL.

**Conclusion:**

VAHL is frequently observed after BNT162b2 administration, more commonly and with higher intensity following the booster dose. To minimize false and equivocal reports in oncological patients, timing of [^18^F]FDG PET-CT should be based on the time intervals found to have a lower incidence of VAHL, and choice of vaccine injection site should be advised, mainly in patients where ASLN are a relevant site of tumor involvement.

## Introduction

A nationwide mass vaccination using the Pfizer BNT162b2 mRNA vaccine against Covid-19 [1] has been initiated in Israel on December 20, 2020, starting with the population over 60 years of age followed by vaccination of all population over the age of 16 [2].

Soon, while reporting [^18^F]FDG PET-CT studies, we identified accumulation of [^18^F]FDG in axillary and supraclavicular lymph nodes (ASLN) which turned out to be ipsilateral to the vaccine injection site. This was not surprising as [^18^F]FDG is not tumor-specific and accumulates in infectious tissue, inflammation, and other hypermetabolic lesions. Increased [^18^F]FDG uptake in regional lymph nodes (LN) has been previously described following vaccination campaign against H1N1 [3, 4] and following vaccination against human papillomaviruses [5].

Several case reports and small cohort studies have already highlighted the medical imaging identification of vaccine-associated lymphadenopathy post-anti-SARS-CoV-2 vaccines [6–18]. Specifically, a very recent special report published by Radiology scientific expert panel explores the concern of accurate imaging report in view of lymphadenopathy associated with vaccination, although, as the authors state, the proportion of patients experiencing some form of lymphadenopathy is not available. The panel suggested that imaging should be postponed to 6 weeks away from vaccination [6]. This recommendation may be problematic in oncologic imaging. A 6-week post-booster vaccine in the case of the Pfizer vaccine, for instance, means 6 weeks plus 3 weeks from the first vaccine. It should be borne in mind that repeated vaccination might be indicated in the future, narrowing the time window for imaging even more.

Despite the 0.3% incidence of post-vaccination lymphadenopathy reported by Polack et al. [1], our impression was that hypermetabolic lymphadenopathy (HLN) incidence identified by [^18^F]FDG PET-CT of vaccinated patients is much higher and may have a direct impact on the diagnostic accuracy of the study in oncologic patients, who are the leading population undergoing PET-CT assessment. We therefore conducted a study on consecutive patients having [^18^F]FDG PET-CT study at the era of mass vaccination aiming to explore the incidence of this finding and to look for ways to reduce its interference when assessing disease extent of oncologic patients on PET-CT. The data on vaccination was available for all patients having a whole-body [^18^F]FDG PET-CT in our department between December 27, 2020, and February 17, 2021.

## Methods

### Patients

A nationwide mass vaccination using the Pfizer–BioNTech BNT162b2 mRNA Covid-19 vaccine against Covid-19 has been initiated in Israel on December 20, 2020 [2]. As of February 17, 2021, a total of 4,157,220 Israeli people received the first dose of the vaccine, and 2,777,825 the booster dose as well, numbers that translate into 44.9% and 30% of the total Israeli population, respectively [19].

After receiving the consent of the institutional ethical committee, all patients over 16 years of age referred for whole-body [^18^F]FDG PET-CT between December 27, 2020, and February 17, 2021, were interviewed regarding the date of the first and booster vaccine doses and the site of injections. Of all [^18^F]FDG PET-CT studies done in the mentioned period, 99 studies were excluded from analysis: 42 due to missing vaccination data, 34 were brain-only studies, 20 studies were of patients younger than 16 years old, and 3 studies were of patients vaccinated in their thigh or buttocks. All Other 951 consecutive patients were interviewed, consisting of the study cohort: 728 vaccinated patients (All-Vac group) and 223 patients that were not vaccinated (control group). Of the vaccinated patients, 346 received the first vaccine only (Vac-1 group), and 382 received both the first and booster vaccine doses (Vac-2 group). Table 1 summarizes the diseases and indications for PET-CT of the study cohort.

**Table 1:** Study population characteristics

	Total (*n* = 951)	All-Vac group (*n* = 728)	Vac-1 group (*n* = 346)	Vac-2 group (*n* = 382)	Control group (*n* = 223)	Pv
Age (years)	68.3 (59.0-75.4)	69.2 (61.1–76.2)	68.3 (57.6–76.5)	70.0 (62.2–76.0)	65.0 (54.1–71.9)	<0.01 ^a,b^
Female	544 (57.2%)	413 (56.7%)	197 (56.9%)	216 (56.5%)	131 (58.7%)	0.86
Lymphoma	163 (17.1%)	113 (15.5%)	53 (15.3%)	60 (15.7%)	50 (22.4%)	0.06
Lung malignancy	150 (15.8%)	124 (17.0%)	63 (18.2%)	61 (16.0%)	26 (11.7%)	0.11
Breast malignancy	143 (15.0%)	113 (15.5%)	54 (15.6%)	59 (15.4%)	30 (13.5%)	0.75
Colorectal/other lower GI malignancy	110 (11.6%)	83 (11.4%)	43 (12.4%)	40 (10.5%)	27 (12.1%)	0.68
Gynecological malignancy	74 (7.8%)	51 (7.0%)	20 (5.8%)	31 (8.1%)	23 (10.3%)	0.14
Genitourinary malignancy	50 (5.3%)	40 (5.5%)	18 (5.2%)	22 (5.8%)	10 (4.5%)	0.79
Pancreatic malignancy	44 (4.6%)	36 (4.9%)	18 (5.2%)	18 (4.7%)	8 (3.6%)	0.67
Head and neck malignancy	42 (4.4%)	35 (4.8%)	18 (5.2%)	17 (4.5%)	7 (3.1%)	0.50
Melanoma/other skin malignancy	41 (4.3%)	32 (4.4%)	16 (4.6%)	16 (4.2%)	9 (4.0%)	0.93
Sarcoma (including GIST)	38 (4.0%)	27 (3.7%)	11 (3.2%)	16 (4.2%)	11 (4.9%)	0.56
Myeloma	34 (3.6%)	26 (3.6%)	10 (2.9%)	16 (4.2%)	8 (3.6%)	0.64
Upper GI malignancy	33 (3.5%)	27 (3.7%)	13 (3.8%)	14 (3.7%)	6 (2.7%)	0.77
Other malignancies	17 (1.8%)	12 (1.6%)	6 (1.7%)	6 (1.6%)	5 (2.2%)	0.83
Non-oncological conditions	12 (1.3%)	9 (1.2%)	3 (0.9%)	6 (1.6%)	3 (1.3%)	0.69
Staging	213 (22.4%)	166 (22.8%)	89 (25.7%)	77 (20.2%)	47 (21.1%)	0.17
Monitor response to therapy	473 (49.7%)	335 (46.0%)	157 (45.4%)	178 (46.6%)	138 (61.9%)	<0.01 ^a,b^
Chemotherapy	283 (29.8%)	191 (26.2%)	90 (26.0%)	101 (26.4%)	92 (41.3%)	<0.01 ^a,b^
Radiotherapy	64 (6.7%)	42 (5.8%)	24 (6.9%)	18 (4.7%)	22 (9.9%)	0.05 ^b^
Biologic therapy	193 (20.3%)	141 (19.4%)	62 (17.9%)	79 (20.7%)	52 (23.3%)	0.29
Immunotherapy	73 (7.7%)	55 (7.6%)	27 (7.8%)	28 (7.3%)	18 (8.1%)	0.94
Recurrent malignancy	105 (11.0%)	89 (12.2%)	42 (12.1%)	47 (12.3%)	16 (7.2%)	0.11
Follow-up with NED	148 (15.6%)	129 (17.7%)	55 (15.9%)	74 (19.4%)	19 (8.5%)	<0.01 ^a,b^
Non-oncological indication	12 (1.3%)	9 (1.2%)	3 (0.9%)	6 (1.6%)	3 (1.3%)	0.69

Categorical variables are reported as frequency and percentage. Continuous variables are reported as median and IQR

^a^a significant difference was found between Vac-1 and control groups

^b^a significant difference was found between Vac-2 and control groups

*GI*, gastrointestinal; *GIST*, gastrointestinal stromal tumor; *NED*, no evidence of disease

### Detection, categorization, and interpretation of regional lymphadenopathy

[^18^F]FDG PET-CT studies were performed on PET-CT scanners (GE Healthcare; DISCOVERY 690 and DISCOVERY MI; 7 to 8 frames; frame time 1.5–3 min) according to our standard protocol with the administration of dilute oral contrast agent, injection of 3.7 MBq/kg [^18^F]FDG approximately 60 min prior to the study. Final PET-CT interpretation was carried out by at least one nuclear medicine specialist with PET-CT experience of at least 8 years.

HLN was recorded when at least one [^18^F]FDG-positive ASLN was identified and reported. HLN was categorized in our data as either tumor nodal involvement (malignant HLN – MHL), benign nodes associated with the vaccine (vaccine-associated HLN – VAHL), or equivocal (equivocal HLN – EqHL). Primary tumor type and site; stage of disease; the presence and location of other abnormal imaging findings, mainly malignant lymphadenopathy in other nodal stations; and findings on previous imaging studies were data assisting in interpreting the nature of the HLN, separating MHL and VAHL. However, if such differentiation could not be obtained, the regional lymphadenopathy ipsilateral to the vaccine injection site was interpreted as EqHL. If no “hot” ASLN was detected, the case was categorized as no-HLN.

In all VAHL identified, the locations of the “hot” nodes identified in the axilla were recorded as axillary level 1, 2, 3 or interpectoral nodes. [^18^F]FDG-uptake intensity was measured in the “hottest” node, using maximal standardized uptake value (SUVmax) calculated as [^18^F]FDG uptake (kBq/mL) divided with the injected dose (MBq) and multiplied with the lean body weight (kg). The size of the largest “hot” node was recorded using short-axis diameter measurement. Enlarged LN were defined as >8 mm for oval and > 10 mm for round LN.

**Fig. 1:**
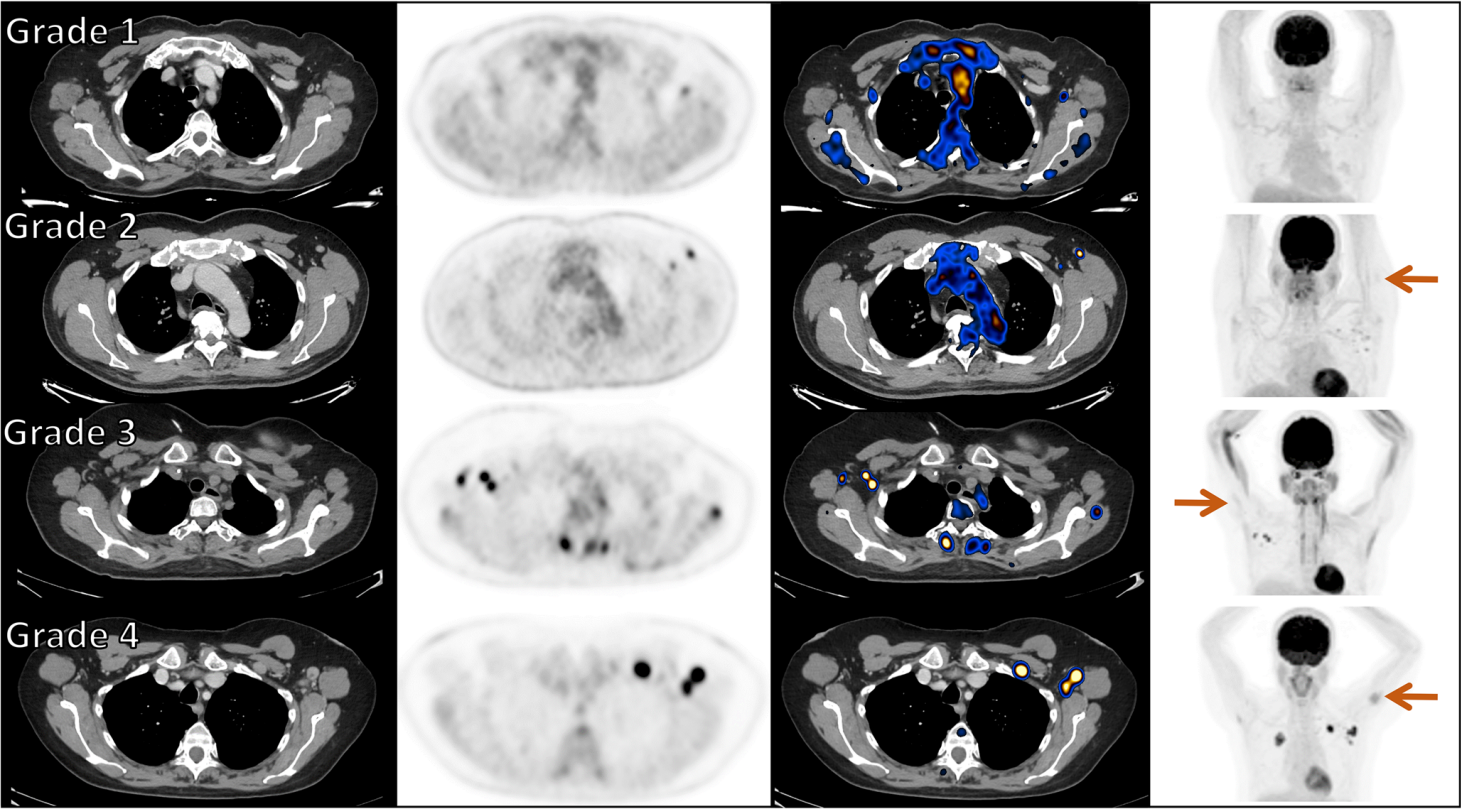
Vaccine-associated hypermetabolic lymphadenopathy (VAHL) grades. Each row represents one patient and includes from left to right CT, PET, and fusion PET-CT trans-axial slices and a maximal intensity projection (MIP) image. From top to bottom: patient referred for staging of colon cancer imaged 9 days following the first vaccine dose, patient referred for follow-up of rectal cancer 13 days following the booster vaccine dose, patient with history of left breast cancer referred for follow-up study 10 days following the first vaccine dose, and a patient referred for staging of right upper lobe lung cancer 1 day following the booster vaccine dose. In all presented cases, HLN was identified in ASLN, attributed to the recent vaccination on the report, and graded in our data based on [^18^F]FDG-uptake intensity. SUVmax measured in the presented cases were 1.97, 3.39, 10.10, and 14.34 from top to bottom. On the bottom row, LN diameter was 14 mm. On the MIP images, brown arrows point hypermetabolism recognized at the vaccine injection site

Based on [^18^F]FDG-uptake intensity and nodal size, VAHL was graded on a 4 grades scale: grade 1, mild [^18^F]FDG-uptake intensity (SUVmax <2.2); grade 2, moderate [^18^F]FDG-uptake intensity (2.2 ≤ SUVmax <4); grade 3, high [^18^F]FDG-uptake intensity (SUVmax ≥4) in normal-size nodes; and grade 4, high [^18^F]FDG-uptake intensity (SUVmax ≥4) in enlarged nodes. Figure 1 illustrates the four different VAHL grades.

### Statistical analysis

Categorical variables were reported as frequency and percentage. Continuous variables were evaluated for normal distribution and reported as median and interquartile range (IQR). Chi-square test and Fisher’s exact test were applied to compare proportions between groups. Independent samples Kruskal–Wallis test and Mann–Whitney test were used to compare continuous variables. Chi-square automatic interaction detection (CHAID) was used to identify a subgroup of patients with similar rates of VAHL grades. All statistical tests were performed using SPSS Statistics Version 27 (IBM, Armonk, NY, USA) and were two-sided, and *p* < 0.05 was considered statistically significant.

## Results

In 332 of 728 (45.6%) vaccinated patients, hypermetabolic ASLN were identified ipsilateral to the vaccine injection site. This finding was found in 36.4% of the patients after the first vaccine and in 53.9% of patients after the booster one. In 17 of the vaccinated patients with HLN (5.1%), the “hot” nodes reflected malignant nodal disease (MHL). In 266 (80.1%) the “hot” nodes were benign nodes associated with the vaccine (VAHL), and in 49 patients (14.8%), the nature of the nodes was equivocal (EqHL) (Table 2).

**Table 2:** Incidence of hypermetabolic lymphadenopathy categories

All-Vac group (*n* = 728)		Vac-1 group (*n* = 346)	Vac-2 group (*n* = 346)	Control group (*n* = 223)	Pv
No HLN	396 (54..4%)	220 (63.6%)	176 (46.1%)	206 (92.4%)	<0.01 ^a,b,c^
HLN	332 (45.6%)	126 (36.4%)	206 (53.9%)	17 (7.6%)	
MHL	17 (2.3%)	10 (2.9%)	7 (1.8%)	17 (7.6%)	<0.01 ^b,c^
EqHL	49 (6.7%)	25 (7.2%)	24 (6.3%)		0.61
VAHL	266 (36.5%)	91 (26.3%)	175 (45.8%)		<0.01

Categorical variables are reported as frequency and percentage

^a^a significant difference was found between Vac-1 and Vac-2 groups

^b^a significant difference was found between Vac-1 and control groups

^c^a significant difference was found between Vac-2 and control groups

### Vaccine-associated hypermetabolic lymphadenopathy

The incidences of VAHL were 36.5%, 26.3%, and 45.8% in All-Vac, Vac-1, and Vac-2 groups, respectively. Table 3 summarizes the grade, location, intensity of uptake, and size of VAHL after the first and after the booster vaccine doses as well as detection of increased uptake in the vaccination site. After the booster vaccine, the incidence of high-intensity VAHL was statistically significantly higher than after the first vaccine, and so was the size of nodes, detection of “hot” nodes beyond level 1 of the axilla, and detection of the vaccination site.

**Table 3:** Comparing VAHL characteristics between Vac-1 and Vac-2 groups

		All- Vac group (*n* = 266)	Vac-1 group (*n* = 91)	Vac-2 group (*n* = 175)	Pv
Grading	Grade 1 VAHL	96 (36.1%)	40 (44.0%)	56 (32.0%)	0.05
	Grade 2 VAHL	112 (42.1%)	40 (44.0%)	72 (41.1%)	0.66
	Grade 3 VAHL	32 (12.0%)	7 (7.7%)	25 (14.3%)	0.12
	Grade 4 VAHL	26 (9.8%)	4 (4.4%)	22 (12.6%)	0.03
Location	Axilla-level 1	264 (99.2%)	90 (98.9%)	174 (99.4%)	>0.99
	Axilla-level 2	100 (37.6%)	24 (26.4%)	76 (43.4%)	0.01
	Axilla-level 3	35 (13.2%)	7 (7.7%)	28 (16.0%)	0.06
	Axilla-interpectoral	39 (14.7%)	5 (5.5%)	34 (19.4%)	<0.01
	Supraclavicular	21 (7.9%)	5 (5.5%)	16 (9.1%)	0.30
Intensity	SUVmax	2.63 (1.85–3.82)	2.40 (1.74–3.19)	2.76 (1.97–4.29)	0.01
Size	Enlarged LN	37 (13.9%)	6 (6.6%)	31 (17.7%)	0.01
	Visible injection site	99 (37.2%)	21 (23.1%)	78 (44.6%)	<0.01

Categorical variables are reported as frequency and percentage; continuous variables are reported as median and IQR

**Fig. 2:**
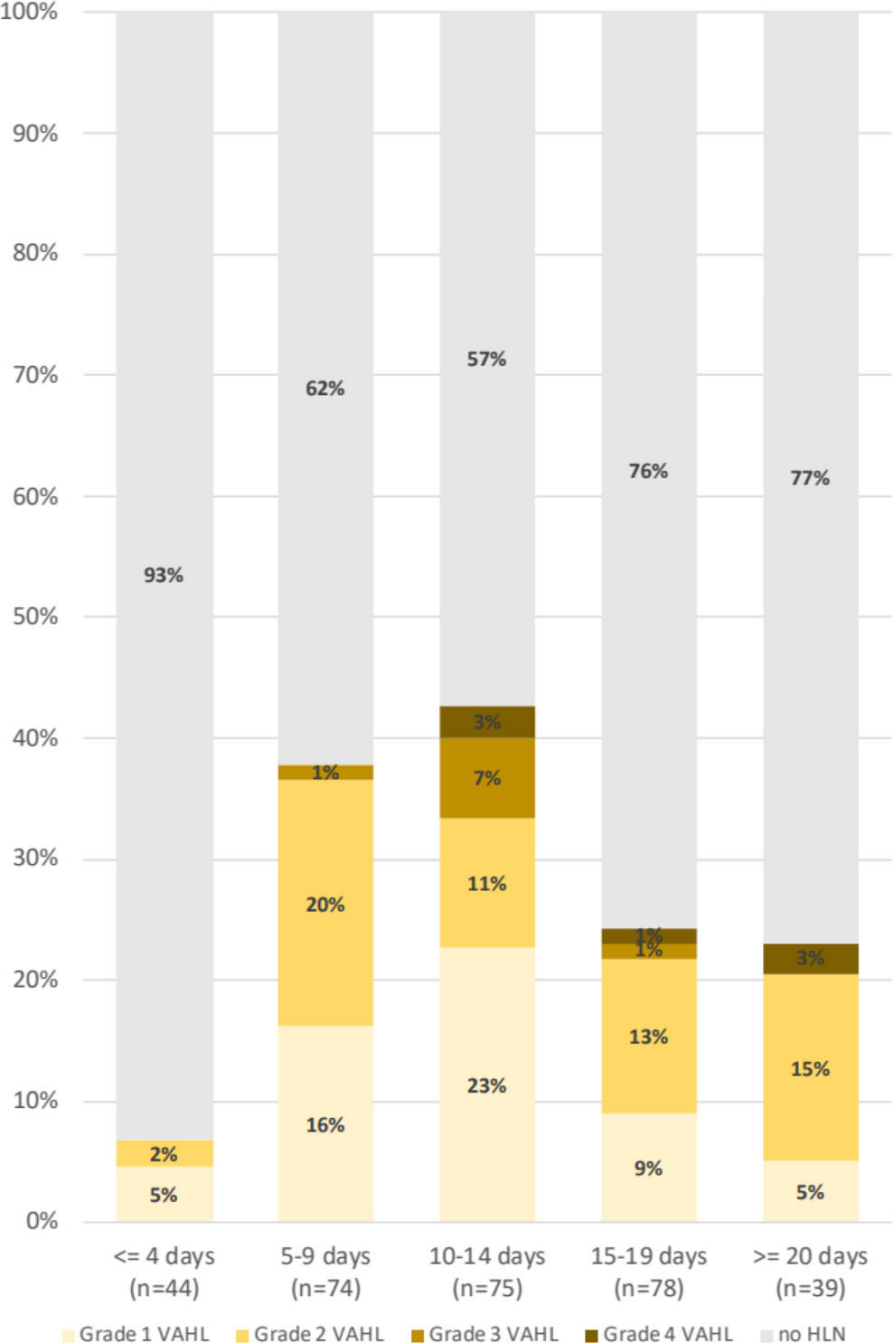
Proportion of vaccinated patients with VAHL and the grade of VAHL in different time points after the first vaccine dose

**Fig. 3:**
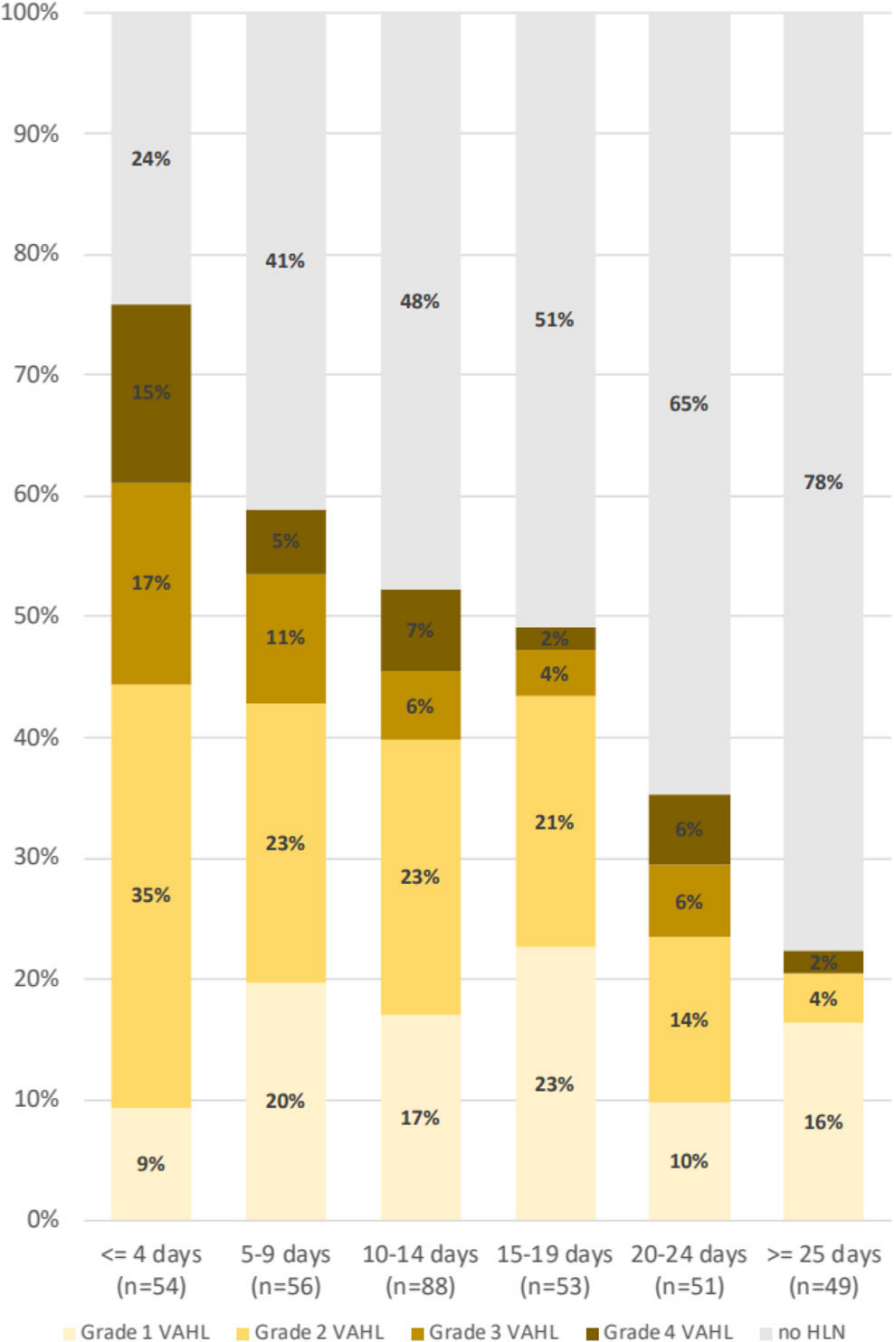
Proportion of vaccinated patients with VAHL and the grade of VAHL in different time points after the booster vaccine dose

Figure 2 illustrates the proportion of vaccinated patients with VAHL and the grade of VAHL at various time points after the first vaccine administration. Using CHAID algorithm, it appears that in the first 5 days and beyond 13 days after the first vaccination, the incidence of VAHL is statistically lower compare to the higher incidence observed 6–12 days after vaccination (Table 4). Figure 3 and Table 5 illustrate that after the booster vaccine, the incidence and grade of VAHL are highest on the first 6 days, decrease gradually over time, and are significantly lower beyond 20 days after vaccination. However, 3 weeks after booster vaccine administration, 29% of vaccinated patients still presented VAHL in our cohort, but only 7% had grade 3 or 4 VAHL.

**Table 4:** Vac-1 group: proportions of VAHL reports in time periods segmented using CHAID algorithm (Pv < 0.01)

	0–5 days (*n* = 54)	6–12 days (*n* = 106)	13+ days (*n* = 150)	Pv
No HLN	50 (92.6%)	56 (52.8%)	113 (75.3%)	<0.01 ^a,b,c^
Grade 1 VAHL	2 (3.7%)	25 (23.6%)	13 (8.7%)	<0.01 ^a,c^
Grade 2 VAHL	2 (3.7%)	20 (18.9%)	18 (12.0%)	0.02 ^a^
Grade 3 VAHL	0 (0.0%)	3 (2.8%)	4 (2.7%)	0.47
Grade 4 VAHL	0 (0.0%)	2 (1.9%)	2 (1.3%)	0.61

Categorical variables are reported as frequency and percentage

^a^a significant difference was found between 0 and 5 days and 6–12 days groups

^b^a significant difference was found between 0 and 5 days and 13+ days groups

^c^a significant difference was found between 6 and 12 days and 13+ days groups

**Table 5:** Vac-2 group: proportions of VAHL reports in time periods segmented using CHAID algorithm (Pv < 0.01)

	0–6 days (*n* = 76)	7–19 days (n = 175)	20+ days (*n* = 100)	Pv
No HLN	18 (23.7%)	87 (49.7%)	71 (71.0%)	<0.01 ^a,b,c^
Grade 1 VAHL	8 (10.5%)	35 (20.0%)	13 (13.0%)	0.11
Grade 2 VAHL	26 (34.2%)	37 (21.1%)	9 (9.0%)	<0.01 ^a,b,c^
Grade 3 VAHL	15 (19.7%)	7 (4.0%)	3 (3.0%)	<0.01 ^a,b^
Grade 4 VAHL	9 (11.8%)	9 (5.1%)	4 (4.0%)	0.07

Categorical variables are reported as frequency and percentage

^a^a significant difference was found between 0 and 6 days and 7–19 days groups

^b^a significant difference was found between 0 and 6 days and 20+ days groups

^c^a significant difference was found between 7 and 19 days and 20+ days groups

**Fig. 4:**
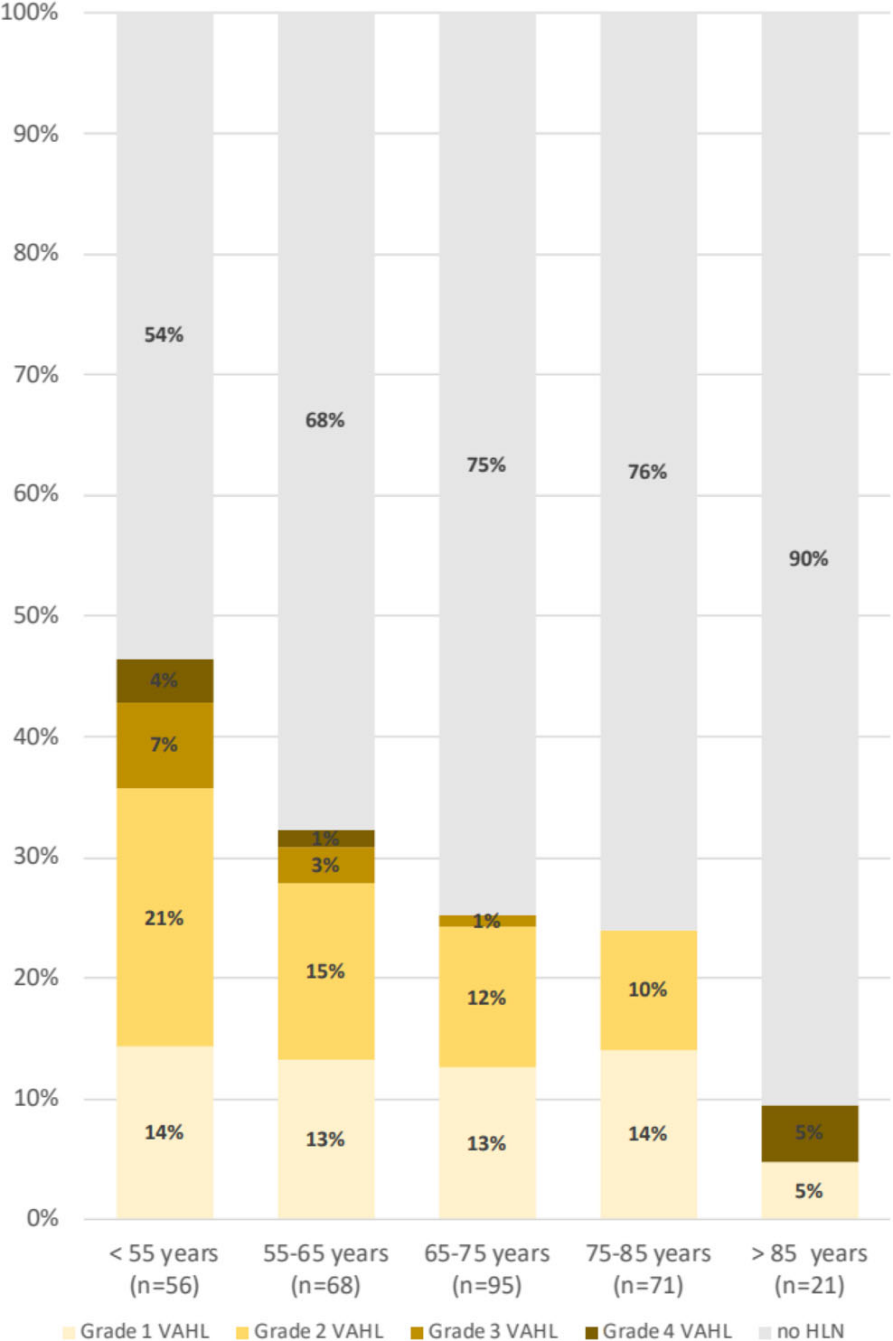
Proportion of vaccinated patients with VAHL and the grade of VAHL in different age groups after the first vaccine dose

**Fig. 5:**
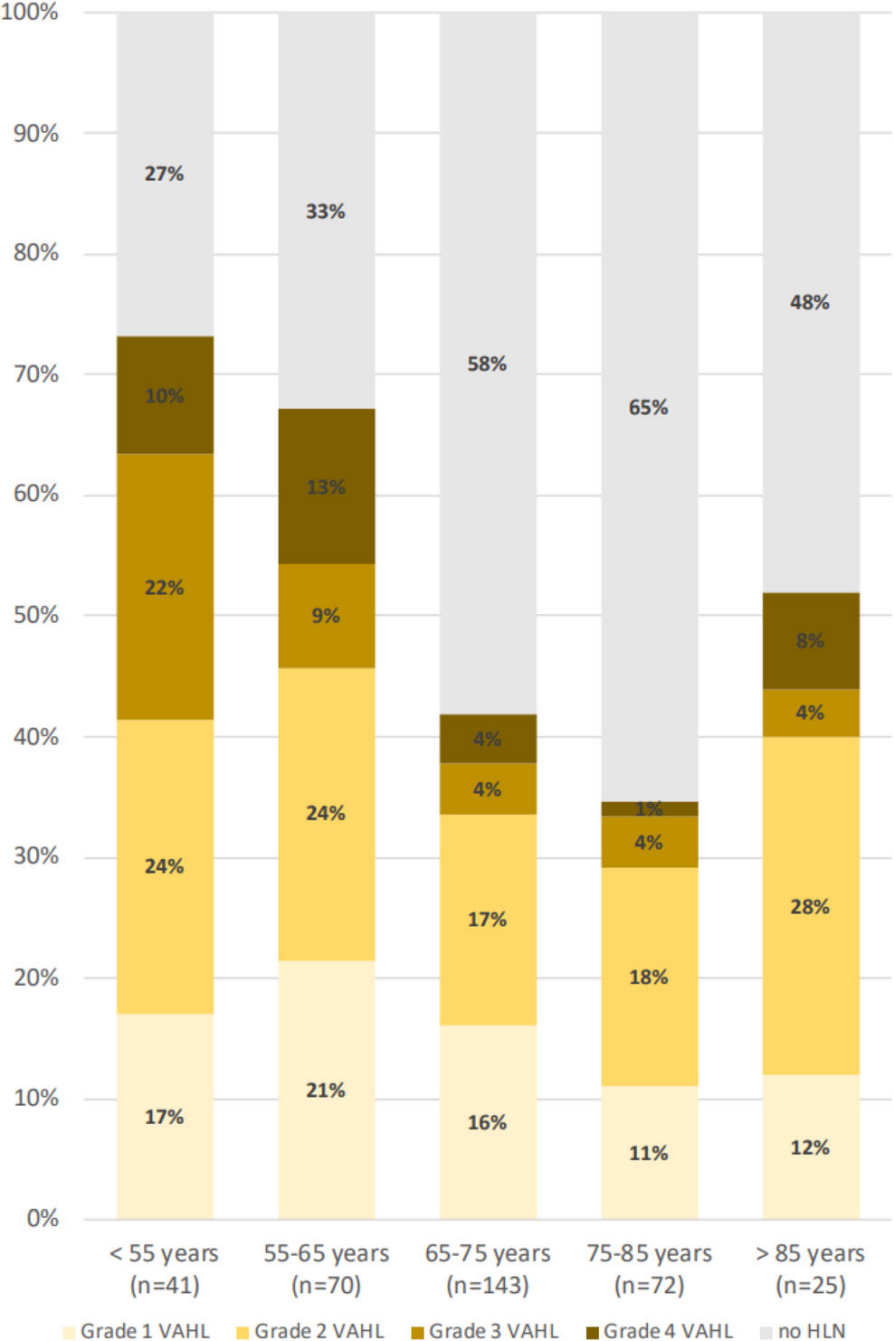
Proportion of vaccinated patients with VAHL and the grade of VAHL in different age groups after the booster vaccine dose

Patients younger than 62 years of age show a higher incidence of VAHL after the first vaccine as well as VAHL of a higher grade (see Fig. 4 and Table 6). Similar results were found after the booster vaccine with 64 years being the age that statistically separates the incidence and grade of VAHL in the different age groups (see Fig. 5 and Table 7). Of note, we found another increase in the incidence of VAHL after the age of 85 in Vac-2 group (Fig. 5).

**Table 6:** Vac-1 group: proportions of VAHL reports in age groups segmented using CHAID algorithm (Pv < 0.01)

	<62.07 (*n* = 93)	>62.07 (*n* = 218)	Pv
No HLN	53 (57.0%)	167 (76.6%)	<0.01
Grade 1 VAHL	15 (16.1%)	25 (11.5%)	0.26
Grade 2 VAHL	17 (18.3%)	23 (10.6%)	0.06
Grade 3 VAHL	5 (5.4%)	2 (0.9%)	0.03
Grade 4 VAHL	3 (3.2%)	1 (0.5%)	0.08

Categorical variables are reported as frequency and percentage

**Table 7:** Vac-2 group: proportions of VAHL reports in age groups segmented using CHAID algorithm (Pv < 0.01)

	<64.35 (*n* = 105)	>64.35 (*n* = 246)	Pv
No HLN	31 (29.5%)	145 (58.9%)	<0.01
Grade 1 VAHL	21 (20.0%)	35 (14.2%)	0.18
Grade 2 VAHL	25 (23.8%)	47 (19.1%)	0.32
Grade 3 VAHL	15 (14.3%)	10 (4.1%)	<0.01
Grade 4 VAHL	13 (12.4%)	9 (3.7%)	<0.01

Categorical variables are reported as frequency and percentage

### PET–CT interpretation in vaccinated patients presenting with hypermetabolic lymphadenopathy in the axilla and supraclavicular region

As demonstrated in Table 2, VAHL was identified and reported in 80.1% of the vaccinated patients presenting with hypermetabolic ASLN. There was no statistically significant difference in the incidence of VAHL when comparing patients with no evidence of disease on PET-CT and those with active malignant disease. The proportions of VAHL reports were not different in patients receiving chemotherapy, radiotherapy, biologic treatment, or immunotherapy.

Malignant hypermetabolic ASLN (MHL) ipsilateral to the vaccination site was interpreted in 5.1% of the vaccinated patients presenting with “hot” nodes in these nodal stations. These patients were either patients with proven nodal disease in these stations or patients with extensive lymphadenopathy mainly above the diaphragm, including the contralateral axilla.

**Fig. 6:**
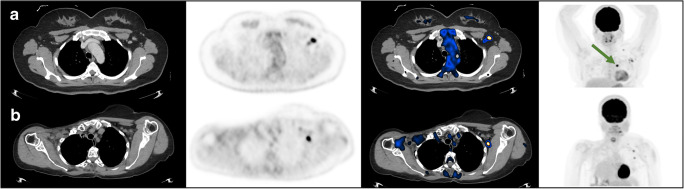
Examples of cases with equivocal reports. Each row represents one patient and includes from left to right CT, PET, and fused PET-CT trans-axial slices and a maximal intensity projection (MIP) image. **a** A patient with newly diagnosed left breast cancer 7 days following the first vaccine dose. Green arrow points the primary tumor. **b** A follow-up study of a patient after resection of sarcoma from the left forearm, imaged 3 days following the booster vaccine dose. In both presented cases, HLN was identified in ASLN, but differentiation between MHL and VAHL could not be obtained, and the lymphadenopathy was reported as equivocal

However, in 49 patients, differentiation between MHL and VAHL could not be made, and the nature of the “hot” LN was considered nonconclusive (EqHL). This group of patients consists of 6.8% of the 719 oncologic patients referred for PET-CT post vaccination and 14.8% of the 332 vaccinated patients presenting with hypermetabolic ASLN ipsilateral to the vaccine injection site. Of the 49 EqHL cases, 20 patients were women with breast cancer ipsilateral to the vaccination arm (eight patients at staging), and 16 were lymphoma patients with nodal disease above the diaphragm. In the remaining 13 patients, the ASLN were relevant lymphatic drainage basins, including patients with upper limb sarcoma, melanoma, and head and neck malignancy with extensive cervical and nodal involvement. Figure 6 illustrates two cases which were interpreted as EqHL.

## Discussion

The Covid-19 pandemic has caused significant morbidity and mortality throughout the world, as well as major social and economic disruptions. In the field of nuclear medicine, some exciting imaging findings associated with Covid-19 were described [20–23].

In late December 2020, soon after the WHO listed the Pfizer–BioNTech BNT162b2 mRNA Covid-19 vaccine as the first to receive emergency validation [24], an Israeli nationwide mass vaccination of the population over the age of 16 has been initiated [2].

Swollen axillary lymph nodes, painful at times, have been described occasionally by vaccinated patients. The clinical trial on the safety and efficacy of the BNT162b2 vaccine reported regional lymphadenopathy in 0.3% of the recipients [1]. The package leaflet for the BNT162b2 vaccine recipient classifies enlarged lymph nodes as an uncommon side effect, stating that it may affect 1 in 100 to 1000 vaccine recipients [25]. PET-CT, however, allows a more objective mode to assess the incidence of lymphadenopathy by detecting “hot” nodes even if of normal size or when located in axillary level 2 and 3 or interpectoral nodal stations that may be overlooked on physical palpation.

Data regarding the date of vaccinations and the side of the arm vaccinated were available in 951 patients having a whole-body [^18^F]FDG PET-CT during the study period, 346 post the first vaccine dose only and 382 post the second booster vaccine as well. “Hot” axillary lymph nodes and occasionally also supraclavicular nodes ipsilateral to the vaccination side were found in 36.4% of patients after the first vaccine and 53.9% after the booster vaccine, revealing the unexpected high incidence of this PET-CT finding, particularly in the setting of mass vaccination of the population. Patients younger than 62–64 years are more prone to show VAHL. The finding of an increase in the incidence of VAHL after the age of 85 in Vac-2 group is unclear and should be interpreted with caution given the small group of such patients.

Assessing the incidence of VAHL on different time points after vaccinations revealed “time windows” which are more suitable for the performance of [^18^F]FDG PET-CT study in vaccinated patients in order to avoid interpretation difficulties due to VAHL. The highest incidence of VAHL after the first vaccination was 6–12 days after vaccination. The incidence is lower in the first 5 days after the first vaccination and in the third week after this vaccination. The booster of the Pfizer vaccine is administered 3 weeks after the first vaccination. Immediately after the booster dose administration, the incidence of VAHL is the highest, decreasing gradually during the next 3 weeks. Even after at least 20 days, as high as 29% of patients will show VAHL; however, only 7% of them show high [^18^F]FDG-intensity uptake (grade 3–4 VAHL). So, it appears that there are three “time windows” where the incidence of VAHL is lower and may be suitable for imaging: the first 5 days after the first vaccine, the third week after the first vaccine (before booster dose is administered), and at least 3 weeks after the booster dose administration.

The high incidence of VAHL in vaccinated patients raises a new challenge in the interpretation of [^18^F]FDG PET-CT of oncologic patients, mainly if the ASLN are a relevant site of malignant lymphadenopathy. The intensity of [^18^F]FDG uptake in VAHL was found to overlap that of malignant nodal involvement, so intensity measurement (SUVmax) cannot be used to differentiate between the two. In routine practice, when “hot” ASLN are detected in vaccinated patients, the reporting physician is making assumptions about the nature of the lymphadenopathy based on tumor type, disease history, previous imaging, etc. Such was the case when PET-CT studies of the study cohort were interpreted. Yet, in 49 patients (14.8% of the vaccinated patients with HLN), differentiation between malignant nodal involvement and VAHL could not be made, and the study was interpreted as equivocal. In some of the patients, this unfortunate nonconclusive report was caused by the fact that vaccines were administrated in the same side as the tumor expected nodal drainage. Therefore, patients with breast cancer, axillary lymphoma, and malignancy of the upper limb (melanoma, for instance) should be advised to be vaccinated in the arm contralateral to the tumor expected nodal drainage.

## Conclusions

Vaccination with BNT162b2 causes [^18^F]FDG-positivity in ASLN ipsilateral to the injection site in high rates, more frequently and with higher intensity after the booster dose. In some cases, and more commonly in patients with breast cancer and axillary lymphoma, recent vaccination with BNT162b2 leads to equivocal reports. Given the high incidence of VAHL identification on [^18^F]FDG PET-CT studies, in the current era of mass vaccination campaigns against SARS-CoV-2, we recommend to include a vaccination anamnesis in all patients before PET-CT scheduling and imaging. Our study also stresses that patients with breast cancer, axillary lymphoma, and malignancy of the upper limb should be advised to be vaccinated in the arm contralateral to the tumor expected nodal drainage. To avoid false-positive and minimize equivocal reports, we suggest to schedule [^18^F]FDG PET-CT for oncological patients either during the first 5 days after the first vaccine dose, during the third week after the first vaccine (before booster dose is administered), or at least 3 weeks after the booster vaccine dose.

## Data Availability

The datasets used and/or analyzed during the current study are available from the corresponding author on reasonable request.

## Abbreviations

HLN: Hypermetabolic lymphadenopathy
VAHL: Vaccine-associated hypermetabolic lymphadenopathy
EqHL: Equivocal hypermetabolic lymphadenopathy
MHL: Malignant hypermetabolic lymphadenopathy
LN: Lymph nodes
ASLN: Axillary or supraclavicular lymph nodes
[^18^F]FDG: F^18^-fluorodeoxyglucose
PET-CT: Positron emission tomography-computed tomography
SUV: Standardized uptake value
MIP: Maximal intensity projection
SARS-CoV-2: Severe acute respiratory syndrome coronavirus 2
Covid-19: Coronavirus disease 2019
